# Drug-Drug Interactions Leading to Tacrolimus Toxicity in a Renal Transplant Patient With COVID-19: The Role of Paxlovid and the Mitigating Use of Phenytoin

**DOI:** 10.7759/cureus.80902

**Published:** 2025-03-20

**Authors:** Ricardo A Pagan Santini, Vinay Nair, Ilan Berlinrut, Gayatri Nair, Madhu Bhaskaran

**Affiliations:** 1 Internal Medicine, Long Island Jewish Forest Hills Hospital, Northwell Health, Forest Hills, USA; 2 Transplant, Northwell Health, Manhasset, USA; 3 Infectious Diseases, Northwell Health, Manhasset, USA; 4 Nephrology and Transplant Nephrology, Northwell Health, Manhasset, USA

**Keywords:** covid 19, general nephrology dialysis and transplanation, pharmacokinetics interactions, protease inhibitors, tacrolimus overdosage

## Abstract

Tacrolimus, a calcineurin inhibitor widely used in transplant immunosuppression, requires careful monitoring due to its narrow therapeutic index and metabolism by cytochrome P450 CYP3A4. We present a case of a 72-year-old kidney transplant recipient who developed acute tacrolimus toxicity following treatment with nirmatrelvir/ritonavir (Paxlovid) for COVID-19. The patient presented with altered mental status, acute kidney injury, and supratherapeutic tacrolimus levels (>90 ng/mL). Given persistent toxicity, tacrolimus was held, and intravenous phenytoin was initiated to enhance clearance through CYP3A4 induction. The patient demonstrated improvement in renal function and tacrolimus levels, allowing for cautious reinitiation of immunosuppression. This case highlights the critical need for vigilant monitoring of drug-drug interactions in transplant recipients and the potential role of phenytoin as an effective therapeutic strategy in managing tacrolimus toxicity induced by CYP3A4 inhibitors as well as the urgent need for developing COVID-19 outpatient treatments with minimal interaction with tacrolimus.

## Introduction

Tacrolimus, a cornerstone in post-transplant immunosuppressive therapy, plays a critical role in maintaining graft viability by suppressing both cellular and humoral immune responses [1]. However, its clinical management presents significant challenges due to its narrow therapeutic index and metabolism via cytochrome P450 CYP3A4, a liver enzyme susceptible to multiple factors, particularly drug-drug interactions. Given these complexities, transplant recipients require meticulous monitoring when initiating new medications that influence CYP3A4 metabolism. 

One of the most clinically significant mechanisms of drug-drug interactions with tacrolimus involves CYP3A4 inhibitors and inducers. CYP3A4 inhibitors will lead to a sharp increase in tacrolimus plasma concentration and subsequent toxicity while CYP3A4 inducers will accelerate drug metabolism, potentially resulting in subtherapeutic tacrolimus levels and increased risk of allograft rejection [2]. 

The COVID-19 pandemic has introduced further complexities in managing drug interactions in transplant patients, particularly with the introduction of antiviral therapies such as nirmatrelvir/ritonavir (Paxlovid) [2]. Ritonavir is a potent CYP3A4 inhibitor, leading to profound increases in tacrolimus levels when co-administered, raising the risk of severe toxicity [2]. Given the necessity of outpatient antiviral treatment in immunosuppressed individuals, strategies to mitigate tacrolimus toxicity are critical. One emerging approach is the use of CYP3A4 inducers, such as phenytoin, to facilitate tacrolimus clearance [2].

In this report, we present the case of a 72-year-old kidney transplant recipient who developed acute tacrolimus toxicity after receiving Paxlovid for COVID-19. The case underscores the importance of understanding pharmacokinetic interactions involving CYP3A4, highlights the clinical consequences of tacrolimus toxicity, and discusses the potential therapeutic role of phenytoin as a CYP3A4 inducer to enhance tacrolimus clearance. This case further emphasizes the urgent need for developing antiviral therapies with minimal drug interaction risks in transplant patients. 

## Case presentation

A 72-year-old male patient with a history of hypertension, type 2 diabetes, hypothyroidism, and a kidney transplant in 2018, with a baseline creatinine of 1.3-1.6 mg/dL, on immunosuppressants (tacrolimus, mycophenolate mofetil, and prednisone) presented via ambulance with altered mental status. At the bedside, the patient was unable to provide his medical history, and collateral information was obtained from his son via phone. The patient came back from India 11 days prior to admission feeling unwell with fevers, nausea, and vomiting. He tested positive for COVID-19 and was prescribed nirmatrelvir/ritonavir (Paxlovid) in the outpatient setting. Hours before presenting to the emergency department, he experienced spiking fever, became confused, failed to follow commands, and had poor oral intake. Initial blood work in the emergency room revealed acute kidney injury, acidosis, and hyponatremia (Table 1). 

**Table 1:** Initial laboratory work on admission GFR: glomerular filtration rate; mmol: millimole; L: liter; mg: miligram; dL: deciliter; ml: mililiter; min: minutes; ng: nanogram

Laboratory	Result	Reference Range
Sodium	126	135-145 mmol/L
Potassium	5.1	3.5-5.3 mmol/L
Chloride	91	96-108 mmol/L
Carbon dioxide	16	22-31 mmol/L
Anion gap	19	5-17 mmol/L
Blood urea nitrogen	39	7-23 mg/dL
Creatinine	2.46	0.50-1.30 mg/dL
GFR	27	>60 ml/min/1.73 m2
Tacrolimus, serum	>90	ng/mL

**Figure 1:**
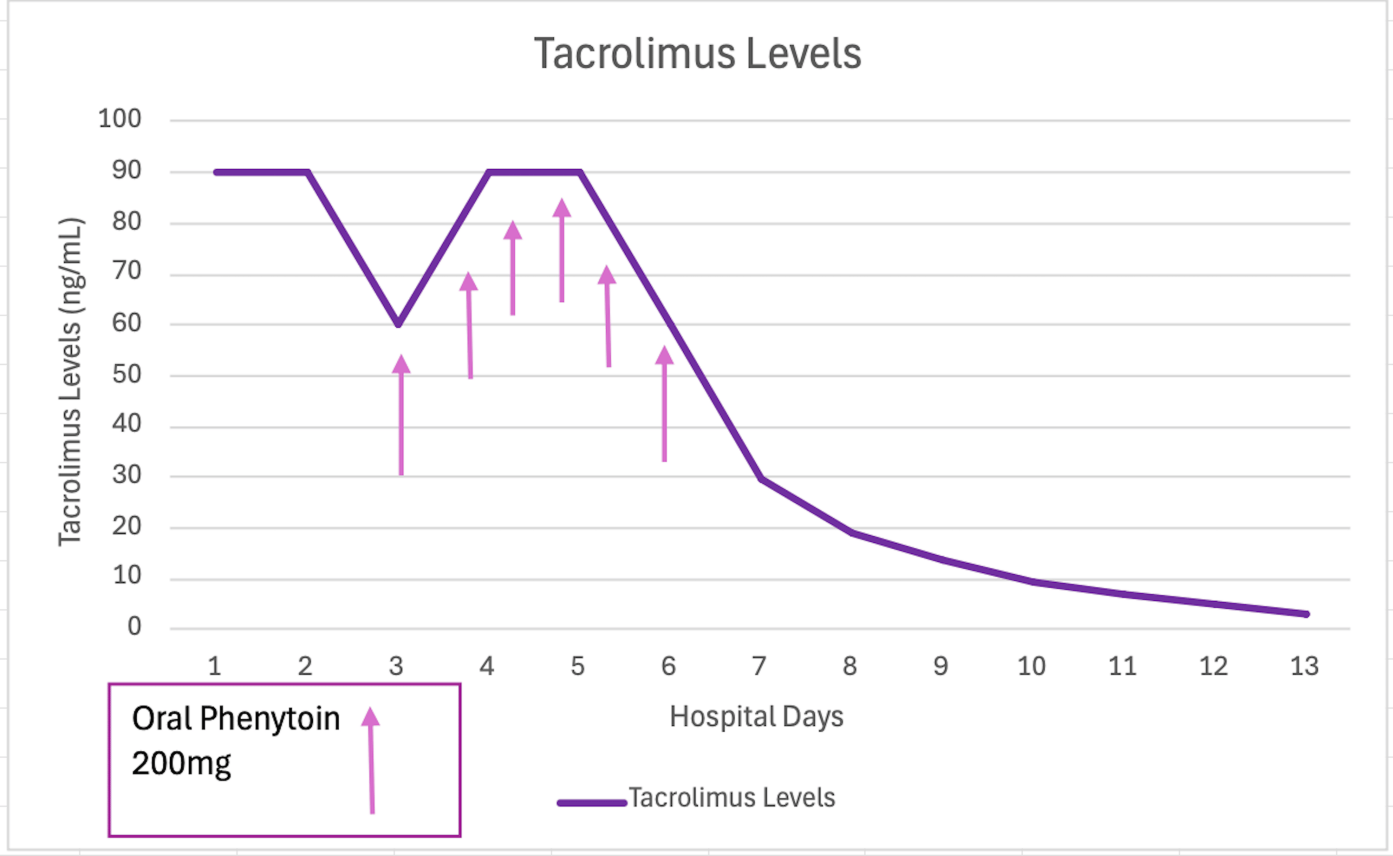
Trend of serum tacrolimus levels through out hospital stay after oral phenytoin

The patient tested positive for COVID-19 and urinalysis revealed pyuria. In the emergency room vancomycin and cefepime were administered. He was admitted as an in-patient and mycophenolate mofetil was held. Tacrolimus level was sent and found to be >90 ng/mL and was subsequently discontinued. Due to persistent elevation of tacrolimus levels (Figure 1), intravenous phenytoin 200mg twice a day was started. The patient received six doses of phenytoin and his tacrolimus levels downtrended to a therapeutic range (Figure 1). In addition, kidney function improved as tacrolimus levels decreased. His mental status returned to baseline, and tacrolimus was resumed at 1 mg once daily in an extended-release form. The patient was discharged home after 13 days of hospitalization. Below is his laboratory test on discharge (Table 2). 

**Table 2:** Laboratory work prior to discharge GFR: glomerular filtration rate; mmol: millimole; L: liter; mg: miligram; dL: deciliter; ml: mililiter; min: minutes; ng: nanogram

Laboratory	Result	Reference range
Sodium	141	135-145 mmol/L
Potassium	4.2	3.5-5.3 mmol/L
Chloride	105	96-108 mmol/L
Carbon dioxide	23	22-31 mmol/L
Anion gap	13	5-17 mmol/L
Blood urea nitrogen	39	7-23 mg/dL
Creatinine	1.33	0.50-1.30 mg/dL
GFR	57	>60 ml/min/1.73 m2
Tacrolimus, serum	2.9	ng/mL

## Discussion

Tacrolimus, initially discovered as a macrolide antibiotic from fungi, is widely used as an immunosuppressant due to its ability to inhibit cell-mediated immune responses [3]. Its mechanism of action involves binding to the immunophilin FK506 binding protein (FKBP12), forming a complex that inhibits calcineurin-induced dephosphorylation of the nuclear factor of activated T cells [4]. This results in the suppression of interleukin-2 (IL-2) transcription, leading to reduced T-cell-mediated activity [1]. Although its mechanism is well understood and very effective, challenges in clinical practice often arise from its pharmacodynamics and metabolism rather than its primary mode of action. Tacrolimus is primarily metabolized by the cytochrome P450 which is a hemeprotein that is classified by gene sequences [4]. In the case of tacrolimus, metabolism is through CYP3A4 which is the most abundant of the CYP enzymes, constituting approximately one-third of the CYP enzymes found in the intestinal lining and the liver [4]. While the pharmacodynamics and primary mechanism of action of tacrolimus are well understood, its clinical management is further complicated by patient-specific factors, such as age, gender, physiological states, and underlying conditions, which significantly influence its metabolism and therapeutic outcomes [4]. 

In the case of pediatric patients, up to two- to fourfold higher doses of tacrolimus are required to maintain therapeutic levels due to physiological factors such as bowel length, hepatic blood flow, and maturation differences in the expression of CYP3A4/3A5 [5]. Conversely, elderly patients often exhibit altered metabolism due to comorbidities, drug-drug interactions, and a higher risk of adverse effects [5]. Gender also plays a role, with females generally requiring higher tacrolimus doses and having a greater susceptibility to side effects [6]. During pregnancy, dose requirements increase by 25-50%, influenced by heightened CYP3A4 activity, increased plasma volume, hypoalbuminemia, and anemia [6]. Disease states further impact tacrolimus metabolism. For instance, inflammatory conditions that lead to alteration of hepatic hemodynamics and upregulation of CRP have been shown to reduce the expression of CYP3A4, while systemic hypoxia has interestingly been shown to increase the expression of this enzyme [6]. Infections such as viral hepatitis can impair tacrolimus metabolism as well, complicating drug management [6]. 

Beyond patient-specific physiological and pathological factors, external influences such as drug interactions and dietary components remain as one of the factors that most modulate the metabolism and therapeutic efficacy of tacrolimus. Drug interactions are largely divided into inhibitors or inducers of this enzyme. With inhibitors, in particular irreversible inhibitors, the effect is more detrimental since the effect lasts longer than with reversible inhibitors due to the formation of metabolic intermediates that bind irreversibly to the enzyme and then inactivate it [7]. The most common examples of medications that have this irreversible effect are clarithromycin, diltiazem, erythromycin, itraconazole, ketoconazole, ritonavir, and verapamil [7]. Reversible inhibitors that are common are estrogen, and antidepressants in particular venlafaxine, nefazodone, sertraline, and fluoxetine [8]. Compared to the inhibitors, inducers of the CYP3A4 have a slower onset in affecting metabolism which leads to a decrease in the effect of the medication [8]. Nevertheless, this can lead to toxicity if the increased metabolism of the parent compound is accompanied by an increase in exposure to a toxic metabolite [8]. Common examples are antiseizure medications phenobarbital and, phenytoin [8]. Additionally, dietary components such as grapefruit, black pepper, and goldenseal can inhibit CYP3A4, whereas high-fat content and high carbohydrates can reduce tacrolimus absorption from the gut [8]. 

The intricate interplay between drug interactions, dietary factors, and tacrolimus metabolism underscores the challenges in optimizing its therapeutic efficacy. When these interactions are not adequately managed, they can lead to significant complications, including tacrolimus toxicity, which requires targeted intervention based on its unique pharmacokinetic properties. Tacrolimus toxicity can manifest as renal failure, neurotoxicity, gastrointestinal disturbances, electrolyte imbalances, and other nonspecific symptoms [9]. Although in clinical practice it is not utilized gastric lavage in theory could be effective if used as an early intervention and this is due to tacrolimus having poor bioavailability in the gastric fluids with low dissolution [9]. Activated charcoal would be ineffective as tacrolimus is highly bound to protein and has minimal biliary excretion [9]. Based on that same principle of high protein binding and sequestration in red blood cells, the use of hemodialysis and plasmapheresis is also ineffective in drug removal and treating acute toxicities [10]. 

As discussed previously, the clearance and metabolism of tacrolimus is largely through the liver, so targeting the CYP3A4 enzyme is an effective way to facilitate clearance. One described way to achieve this is to utilize medications that induce the CYP3A4 enzyme. One of the most used agents is phenytoin, an anticonvulsant agent that works by blockading the voltage-dependent membrane sodium channels responsible for increasing the action potential and thus preventing the spread of the seizure focal point [10]. This medication can assist with the clearance of tacrolimus and also help with seizure prevention secondary to drug toxicity. Phenytoin’s effect on CYP3A4 is particularly pronounced in the central-lobular hepatocytes. The induction of CYP3A4 enzyme in this area promotes faster clearance of tacrolimus with fewer side effects as compared to metabolism in peripheral hepatocytes where it could take up to two weeks for medication clearance [10]. 

Other combinations of medications that have been attempted in conjunction with phenytoin include the use of methylprednisone. Methylprednisolone is a weak CYP3A4 inducer, however, its combined efficacy remains inadequately studied [11]. It is also important to factor in the duration of treatment of phenytoin. Studies have shown that duration of administration of phenytoin can variably affect cytochrome p450 [11]. Phenytoin administration for 2-4 days increases the expression of certain cytochrome P450 enzymes, whereas extended use (>8 days) decreases the expression of other enzymes of the p450 family [12]. The route of administration of phenytoin has also been shown to influence outcomes. For instance, the use of intravenous phenytoin has been shown to provide a faster way to clear tacrolimus compared to oral phenytoin [12]. 

While traditional approaches to managing tacrolimus toxicity focus on its pharmacokinetic properties and interactions with established medications, the emergence of new antiviral therapies during the COVID-19 pandemic has introduced additional challenges. Protease inhibitors containing medications like nirmatrelvir/ritonavir (Paxlovid) have further underscored the critical role of CYP3A4 modulation in maintaining tacrolimus efficacy and safety. Nirmatrelvir/ritonavir is used to treat mild to moderate COVID-19 in the first five days of illness [1]. Nirmatrelvir is a peptidomimetic inhibitor of the SARS-CoV-2 main protease which leads to the inhibition of viral replication while ritonavir a protease inhibitor, inhibits the metabolism of nirmatrelvir leading to increased plasma concentrations [1]. The mechanism that ritonavir has this effect is by inhibiting the CYP3A4 enzyme [1]. Historically protease inhibitors have been used in this novel fashion to boost levels of HIV anti-retroviral therapy. Due to known interactions of protease inhibitors and CYP3A4, there have been studies following HIV patients with transplants taking tacrolimus and simultaneously on protease inhibitors [13]. The studies performed have shown that different protease inhibitors exhibit different interactions with tacrolimus. For example, nelfinavir has demonstrated the highest inhibition of the CYP3A4 [13]. Also, It has also been demonstrated that the withdrawal of different protease inhibitors affected the CYP3A4 in different timeframes [13]. Of note, it was also shown that the effect of protease inhibitors on tacrolimus was affected by which organ was transplanted, for instance, there was more inhibition of the CYP3A4 in liver transplant patients than in kidney patients [14]. Despite no guidelines on how tacrolimus should be dosed with the use of protease inhibitors it is suggested that there should be an empirical reduction or temporary stoppage of calcineurin inhibitor (CNI) administration upon starting Paxlovid [14]. Also, subsequent CNI dosing should be guided by trough drug levels for the duration of Paxlovid treatment [14]. After completing Paxlovid therapy, resuming the original CNI dose is reasonable; however, monitoring trough levels for an additional 1-2 days is advisable [15]. 

While nirmatrelvir/ritonvir has become the preferred outpatient antiviral therapy due to its demonstrated effectiveness, other options such as molnupiravir have also been explored, albeit less frequently. Unlike Paxlovid, which combines protease inhibitors to modulate viral replication and has significant interactions with CYP3A4, molnupiravir offers a different mechanism of action with fewer documented effects on CYP3A4. This distinction has prompted investigations into alternative therapeutic strategies to mitigate drug interactions, particularly in transplant patients requiring immunosuppressive therapies like tacrolimus. Molnupiravir tends to be used less frequently due mostly to its limited effectiveness in preventing hospitalization compared to Paxlovid [16]. It is a prodrug of the nucleoside derivative N-hydroxycytidine, targeting the viral RNA-dependent RNA polymerase [16].

Historically, antiviral monotherapies have been found to be less effective than combination therapies due to the synergism exhibited by combination therapies and protease inhibitors [17]. Alternative options have been explored to minimize drug interactions involving CYP3A4 with one such approach combining molnupiravir and other protease inhibitors. These combination therapies have shown synergistic antiviral activity against COVID-19, however, no studies of pharmacodynamics and effects on the cytochrome p450 compared to nirmatrelvir/ritonvir have been done [17]. Based on this, agents that have the least interaction with CYP3A4 should be researched as alternatives for transplant patients who are on immunosuppressive medications that interact with CYP3A4. 

## Conclusions

Tacrolimus, an essential immunosuppressant for transplant recipients, presents significant challenges in maintaining therapeutic levels because it is metabolized by the CYP3A4 enzyme, which is influenced by various factors, particularly drug interactions. The COVID-19 pandemic introduced additional complexities to transplant recipients that require antiviral therapy such as nirmatrelvir/ritonavir (Paxlovid), which interacts with tacrolimus through CYP3A4 inhibition of ritonavir and can potentially cause toxicity. The presented case underscores the importance of monitoring drug interactions constantly in transplant recipients for potential toxicity and the use of therapeutic strategies such as CYP3A4 inducers, including phenytoin, to mitigate toxicity risks. Phenytoin has demonstrated effectiveness in enhancing tacrolimus clearance, though its administration requires careful consideration of duration and route. While alternative antiviral monotherapies therapies without protease inhibitors have been trialed, their efficacy has been very limited. Another potential approach involves utilizing protease inhibitors with minimal interaction or inhibitory effects on CYP450 enzymes ensuring safer use in transplant recipients but also reducing the risk of adverse interactions with other medications. In the interim, tailored therapeutic strategies that account for patient-specific factors and updated medication interaction profiles remain essential to optimize outcomes in transplant patients facing complex pharmacokinetic challenges. 
